# Risk of bias judgments for random sequence generation in Cochrane systematic reviews were frequently not in line with Cochrane Handbook

**DOI:** 10.1186/s12874-019-0804-y

**Published:** 2019-08-05

**Authors:** Ognjen Barcot, Matija Boric, Tina Poklepovic Pericic, Marija Cavar, Svjetlana Dosenovic, Ivana Vuka, Livia Puljak

**Affiliations:** 1 Department of Surgery, University Hospital Split, Spinciceva 1, Split, Croatia; 2 Department for Research in Biomedicine and Health, University of Split, School of Medicine, Soltanska 2, Split, Croatia; 3 Department of Radiology, University Hospital Split, Spinciceva 1, Split, Croatia; 4 Department of Anesthesiology and Intensive Care, University Hospital Split, Spinciceva 1, Split, Croatia; 5 Center for Evidence-Based Medicine and Health Care, Catholic University of Croatia, Ilica 242, 10000 Zagreb, Croatia

**Keywords:** Risk of bias, Cochrane, Systematic reviews, Randomisation, Sequence generation, Selection bias

## Abstract

**Background:**

Assessing the risk of bias (RoB) in included studies is one of the key methodological aspects of systematic reviews. Cochrane systematic reviews appraise RoB of randomised controlled trials (RCTs) with the Cochrane RoB tool. Detailed instructions for using the Cochrane RoB tool are provided in the Cochrane Handbook for Systematic Reviews of Interventions (The Cochrane Handbook). The purpose of this study was to analyse whether Cochrane authors use adequate judgments about the RoB for random sequence generation of RCTs included in Cochrane reviews.

**Methods:**

We extracted authors’ judgments (high, low or unclear RoB) and supports for judgments (comments accompanying judgments which explain the rationale for a judgment) for random sequence generation of included RCTs from RoB tables of Cochrane reviews using automated data scraping. We categorised all supporting comments, analysed the number and type of various supporting comments and assessed adequacy of RoB judgment for randomisation in line with recommendations from the Cochrane Handbook.

**Results:**

We analysed 10,103 RCTs that were included in 704 Cochrane reviews. For 5,706 RCTs, randomisation was not described, but for the remaining RCTs, it was indicated that randomisation was performed using computer/software/internet (*N* = 2,850), random number table (*N* = 883), mechanical method (*N* = 359) or it was incomplete/inappropriate (*N* = 305).

Overall, 1,220/10,103 trials (12%) did not have a RoB judgment in line with Cochrane Handbook guidance about randomisation. The highest proportion of misjudgements was found for trials with high RoB (28%), followed by those with low (20%) or unclear (3%). Therefore, one in eight judgments for the analysed domain in Cochrane reviews was not in line with Cochrane Handbook, and one in four if the judgment was "high risk".

**Conclusion:**

Authors of Cochrane reviews often make judgments about the RoB related to random sequence generation that are not in line with instructions given in the Cochrane Handbook, which compromises the reliability of the systematic reviews. Our results can help authors of both Cochrane and non-Cochrane reviews which use Cochrane RoB tool to avoid making common mistakes when assessing RoB in included trials.

**Electronic supplementary material:**

The online version of this article (10.1186/s12874-019-0804-y) contains supplementary material, which is available to authorized users.

## Background

Randomised controlled trials (RCTs) are considered crucial for objectively testing the efficacy and safety of interventions [1]. They are often used to inform clinical practice and included in systematic reviews to obtain an even higher level of evidence through evidence synthesis. However, a recent systematic review of meta-epidemiological studies indicated that estimates about effects of interventions might be exaggerated in RCTs with inadequate or unclear sequence generation and blinding [2].

Therefore, RCTs can be biased if flaws in their design and conduct lead to overestimation or underestimation of intervention effect estimates. Because of this potential for bias, assessment of the risk of bias (RoB) in trials is one of the usual methodological steps during the preparation of Cochrane systematic reviews. Bias has been defined as any systematic error that can negatively impact estimated effects of interventions and lead to incorrect conclusions about the efficacy and safety of analysed interventions [3].

In Cochrane’s RoB tool, systematic review authors are expected to provide judgment about the level of RoB as low, unclear or high for seven different potential domains of bias: selection bias (biased allocation to interventions) due to inadequate generation of a randomised sequence; selection bias (biased allocation to interventions) due to inadequate concealment of allocations prior to assignment; performance bias due to knowledge of the allocated interventions by participants and personnel during the study; detection bias due to knowledge of the allocated interventions by outcome assessors; attrition bias due to amount, nature or handling of incomplete outcome data; reporting bias due to selective outcome reporting; and other bias due to problems not covered elsewhere. Cochrane authors should provide their RoB assessments in the RoB table, where the assessment for each domain needs to contain two sections: a judgment (i.e., the risk is low, unclear or high) and an accompanying supporting comment that needs to justify the judgment. The first domain analysed in the Cochrane RoB tool is assessing the process of generating a randomisation sequence as a part of the assessment of selection bias [4].

A recent report analysed the evolution of reporting and inadequate methods over time in 20,920 RCTs included in Cochrane reviews [5]. The authors analysed data from RCTs included in all Cochrane reviews published between March 2011 and September 2014, which reported an evaluation of the Cochrane RoB items, including sequence generation. The results indicated that unclear risk for sequence generation was found in 49% of trials, high risk in 4% of all trials and low risk in 48% of trials included in the analysed Cochrane reviews [5]. Additionally, it was found that the proportion of trials with unclear RoB for sequence generation decreased over time, falling from 69% in the 1986–1990 cohort of trials to 31% in the 2011–2014 cohort. The proportion of trials with high RoB for sequence generation fell from 4.6% in 1986–1990 to 3.2% in 2011–2014 [5].

However, it is possible that the way the Cochrane authors judge RoB for sequence generation is highly variable. We have already proved this for RoB domains for allocation concealment [6], blinding of participants and personnel [7], incomplete outcome data [8], selective reporting [9], and other bias [10]. In that case, results presented in the study of Dechartres et al. [5] or similar studies would not be based on consistent ratings of RoB in Cochrane reviews, and improvements shown for certain RoB domains could be misleading.

The objectives of this study were two-fold: to evaluate the rationales based on which judgments related to random sequence generation were made for trials in Cochrane reviews, and to investigate the proportion of inadequate judgments about randomisation based on independent re-assessments using guidance from the Cochrane Handbook.

## Methods

### Study design

This was a methodological study that analysed methods of published Cochrane reviews.

### Inclusion and exclusion criteria

**Fig. 1:**
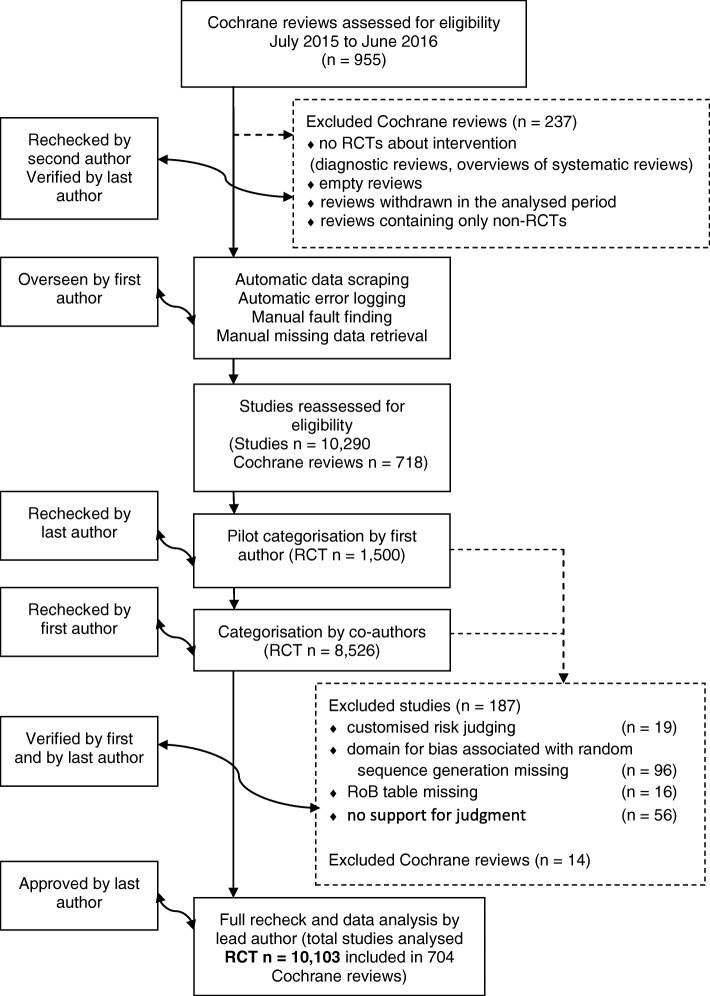
Inclusion and exclusion of studies. *RCT* randomised controlled trial, *RoB* risk of bias

We retrieved Cochrane reviews of RCTs from The Cochrane Library via advanced search limited to interventions published from July 2015 to June 2016 (*N* = 955). We used the advanced search option because it enables the use of search limits such as content type, publication date, etc. We excluded all Cochrane reviews (*N* = 237) that did not include RCTs about interventions (diagnostic Cochrane reviews, overviews of systematic reviews), as well as empty reviews, which only contained non-RCTs and reviews that were withdrawn in the analysed period (Fig. 1). If a Cochrane review included both randomised and non-randomised trials, we analysed the RoB table for included RCTs only.

### Screening for study eligibility

One author assessed all titles and abstracts to establish the eligibility of Cochrane reviews for inclusion. The second author verified the assessments of the first author.

### Outcomes of this study

We analysed the number and type of various supporting comments for the Cochrane RoB domain of random sequence generation. We also analysed the adequacy of judgments for this domain, in line with recommendations from the Cochrane Handbook. The Handbook was used as a gold standard in our assessment. If the judgments of Cochrane authors were contrary to the guidance from the Cochrane Handbook, we considered them inadequate.

### Methodological approach

To analyse adequacy of judgments of Cochrane authors regarding RoB related to randomization, we have used four methodological steps. First, we conducted automatic data extraction from Cochrane RoB tables in which judgments and comments of Cochrane reviews are located. Second, we categorized comments of Cochrane authors based on instructions from Cochrane Handbook. Third, we compared whether judgments of Cochrane authors (RoB is high, unclear or low) were adequately explained by supporting comments. Fourth, we made conclusions based on adequacy of judgments of Cochrane authors regarding RoB associated with random sequence generation.

### Data extraction

Data extraction was automated in a stepwise manner in Microsoft Excel 2010 (Microsoft, Redmond, WA, USA) using macro-commands written in Visual Basic for Applications (VBA, Microsoft, Redmond, WA, USA) by the first author (OB). Data scraping was done by automated copying of all the content from The Cochrane Library webpage for every eligible Cochrane review to a separate spreadsheet in MS Excel. RoB tables were extracted from raw data for every study included in a review by a series of macro-commands (see Additional files 1 and 2). Automatic data extraction from Cochrane reviews was facilitated by the uniform organisation of webpages in which each review was published. In the section ‘Characteristics of included studies’ for each study, first a unique identifier is indicated, then the table with characteristics of a study, followed by a RoB title with the subtitle ‘Risk of bias’.

The data extraction process required programming and testing at every step to avoid errors and flag missing data. Coded algorithms were applicable for this purpose only and cannot be reused to extract other data without reprogramming. Initial testing of the data extraction indicated several challenges. We noticed that some predefined RoB domains were missing. For this reason, we extracted RoB tables in their original appearance, as they were prepared by Cochrane authors, and checked and sorted manually. Missing domains were logged by error handling subs and checked manually. For the purpose of this study, an Excel spreadsheet was generated containing all the details of the random sequence generation domain. This was achieved by automated extraction from the corresponding RoB table for every included study. Additionally, to control for the possibility that Cochrane authors did not create a RoB table for each included study, we extracted the number of included studies from the text too, to double-check the number of RoB tables that were supposed to be present in the Cochrane review.

### Calibration of categorisations

First, 1,500 trials were analysed by the first author (OB) against categories provided in the Cochrane Handbook, which was verified by the last author (LP). This calibration exercise was used to create a spreadsheet with drop-down menus that included pre-determined categories. After calibration, for each trial included in analysed Cochrane reviews, we categorized each supporting comment for RoB related to randomization, using those pre-determined categories. Categorisations of comments from remaining trials were conducted by four persons (MB, TPP, MC, SD), and then verified by the OB. All authors involved in categorisations were medical professionals familiar with the Cochrane RoB tool.

### Categorisations

**Fig. 2:**
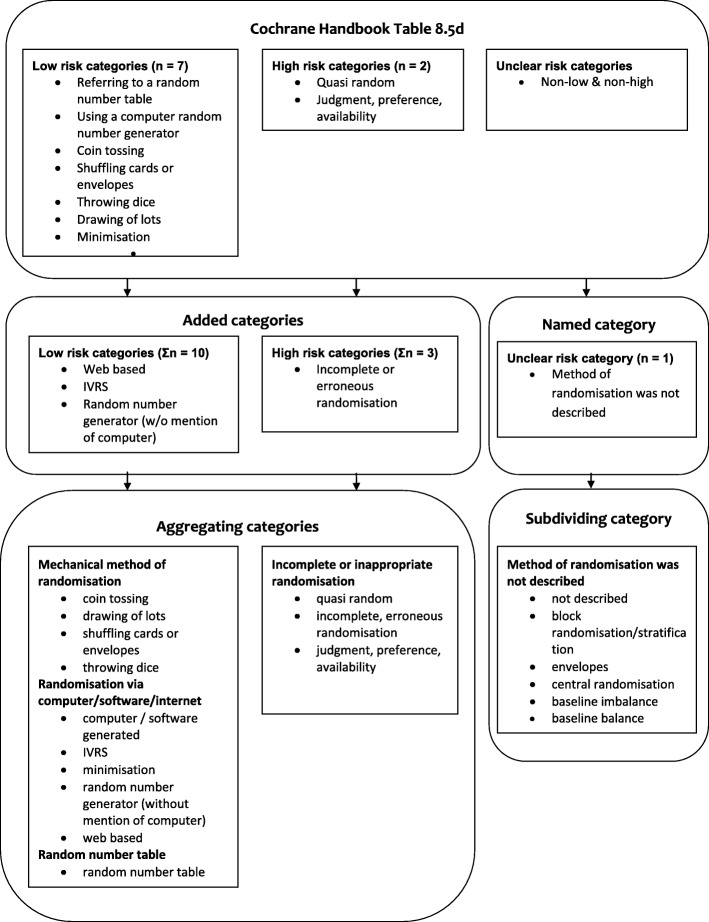
Categorisation, subcategorisation, and aggregation. w/o = without

To categorise supports for judgments, we used recommendations from the Cochrane Handbook’s Table 8.5.d: Criteria for judging the RoB in the ″Risk of bias″ assessment tool. We categorised all the supports for judgments into categories depending on the comment that the Cochrane authors provided to explain their judgment (Fig. 2).

### Categories for trials judged with low risk of bias

For the low risk of bias judgment, we used all seven examples from this tool as independent categories supporting the judgment (random number table, computer random number generator, coin tossing, shuffling cards or envelopes, throwing dice, drawing of lots and minimisation). Additionally, we created a separate category for comments stating a webpage was used as a method of randomisation where an HTTP address was cited and one for an Interactive Voice Response System (IVRS). If the Cochrane authors only wrote in support for a judgment that a ″random number generator″ was used, without mentioning the computer, this was put in a separate category – “random number generator (without mention of computer)”.

### Categories for trials judged with high risk of bias

According to the Cochrane Handbook, two categories support high risk judgment: quasi-random for every type of randomisation using some form of systematic non-random component and randomisation according to the judgment of a physician, preference of a subject or availability of intervention. We added another high risk judgment category – incomplete or erroneous randomisation when comments indicated that randomisation was partial or flawed in any way.

### Categories for trials judged with unclear risk of bias

For every comment not permitting categorisation to any of the 13 high or low risk comment categories, we created a new category – method of randomisation was not described. This included all the instances when the Cochrane authors indicated that something was not described or have only indicated that the study was described as randomised, without mentioning a method of randomisation.

″Method of randomisation was not described″ category was also used when, in supporting judgments, we found descriptions such as: *no information*, *no information available*, *not described*, *not stated*, *not reported*, *unreported* and any similar descriptions. In such cases, we assumed that the review authors wanted to say that the method of randomisation was not described. Similarly, the ″method of randomisation was not described″ category was used if the Cochrane authors wrote only that information about randomisation was on a certain page/table/figure, but no details of what was written there, only comments such as, *assumed*, *same as above, as above, see previous, see XY study, appendix*, *used CONSORT flow diagram*, *Chinese article, translation required*, *trial was stopped*, *not adequately designed*, *study withdrawn prior to enrollment*.

Further on, the following supporting comments were categorised as ″method of randomisation was not described″ if the support for judgment mentioned only baseline characteristics of participants, block randomisation or stratification, randomisation by envelopes, central randomisation by statistics department, pharmacy or a third party without a description of a method used. Based on these supporting comments we created six subcategories in the ″method of randomisation was not described″ parent category. Some of these supporting comments did indicate certain aspects of the methodology associated with the random sequence generation, but not sufficiently enough to be properly judged.

Thus, we created a total of 19 categories of supports for judgment, which were grouped into the following 5 parent categories: i) random number table, ii) randomisation via computer/software/internet that presumes use of electronic automation as in IVRS or random number generation or use of complex algorithms as in minimisation, iii) mechanical method of randomisation such as coin tossing, drawing of lots, shuffling cards or envelopes and throwing dice, iv) incomplete or inappropriate randomisation and v) method of randomisation was not described.

### Statistics

We used frequencies and percentages to present descriptive data.

## Results

We analysed 10,290 RCTs included in 718 Cochrane reviews. During analysis, we had to exclude 187 RCTs and 14 reviews (Fig. 1). We excluded 19 RCTs due to customised risk judging (e.g. risk of bias judged as “moderate”). An additional 112 trials were excluded because of missing RoB table or domain associated with random sequence generation. There were 49 RCTs in which we found no specific reason for the missing RoB or domain, while 54 trials were not randomised, three webpage errors caused duplicate entries and six studies only had abstracts (Table 1). We also excluded 56 RCTs with the empty field or N/A (representing “not available”) in the supporting comment cell of the RoB table.

**Table 1:** Studies missing domain for RoB associated with random sequence generation

Reason for exclusion (study design)	N
Customised risk judging*	19
Domain missing in RoB table	96
NRS†	50
Parallel randomised trial	10
RCT	36
Missing RoB table	16
NRS	4
RCT	3
Duplicate entry	3
Study design unclear - abstract only	6
Total	131

*e.g. “moderate risk of bias”. †NRS = non-randomised study

### Number and type of various supporting comments

In our main analysis, we categorised support for judgment in the remaining 10,103 RCTs into five parent categories. By using criteria from the Cochrane Handbook, the method of randomisation was not described for more than half of those RCTs (*N* = 5,706), while the remaining supporting comments indicated that the randomisation was completed using computer/software/internet (*N* = 2,850), a random number table (*N* = 883), a mechanical method of randomisation (*N* = 359) and incomplete or inappropriate randomisation (*N* = 305). The frequency of these categories and different types of supporting comments, which we found in each of these five categories, are presented in Table 2.

**Table 2:** Supporting comments for judgment of risk of bias regarding randomisation

Categories of supporting comments for judgments of bias associated with the generation of randomisation sequence	Total, N (%)	High, N (%)	Unclear, N (%)	Low, N (%)
Random number table (low risk†)	883 (8.7)	11 (1.2)	31 (3.5)	841 (95.2)
Random number table	883 (100.0)	11 (1.2)	31 (3.5)	841 (95.2)
Randomisation via computer/software/internet (low risk†)	2850 (28.2)	2 (0.1)	36 (1.3)	2812 (98.7)
Computer/software generated	2459 (86.3)	0 (0.0)	17 (0.7)	2442 (99.3)
IVRS*	60 (2.1)	0 (0.0)	7 (11.7)	53 (88.3)
Minimisation	103 (3.6)	2 (1.9)	8 (7.8)	93 (90.3)
Random number generator (without mention of computer)	98 (3.4)	0 (0.0)	0 (0.0)	98 (100.0)
Web based	130 (4.6)	0 (0.0)	4 (3.1)	126 (96.9)
Mechanical method of randomisation (low risk†)	359 (3.6)	7 (1.9)	37 (10.3)	315 (87.7)
Coin tossing	85 (23.7)	0 (0.0)	1 (1.2)	84 (98.8)
Drawing of lots	201 (56.0)	4 (2.0)	12 (6.0)	185 (92.0)
Shuffling cards or envelopes	60 (16.7)	3 (5.0)	24 (40.0)	33 (55.0)
Throwing dice	13 (3.6)	0 (0.0)	0 (0.0)	13 (100.0)
Incomplete or inappropriate randomisation (high risk†)	305 (3.0)	264 (86.6)	32 (10.5)	9 (3.0)
Quasi random	238 (78.0)	209 (87.8)	22 (9.2)	7 (2.9)
Incomplete, erroneous randomisation	41 (13.4)	34 (82.9)	7 (17.1)	0 (0.0)
Judgment, preference, availability	26 (8.5)	21 (80.8)	3 (11.5)	2 (7.7)
Method of randomisation was not described (unclear risk†)	5706 (56.5)	81 (1.4)	4651 (81.5)	974 (17.1)
Not described	4586 (80.4)	69 (1.5)	4272 (93.2)	245 (5.3)
Block randomisation/stratification	679 (11.9)	2 (0.3)	219 (32.3)	458 (67.5)
Envelopes	247 (4.3)	3 (1.2)	117 (47.4)	127 (51.4)
Central randomisation (statistics department, pharmacy, third party)	124 (2.2)	1 (0.8)	0 (0.0)	123 (99.2)
Baseline imbalance between groups	41 (0.7)	6 (14.6)	35 (85.4)	0 (0.0)
Baseline balance between groups	29 (0.5)	0 (0.0)	8 (27.6)	21 (72.4)
Total, N (%)	10103 (100.0)	365 (3.6)	4787 (47.4)	4951 (49.0)

*IVRS = Interactive Voice Response System. †Category RoB judgment according to Cochrane Handbook

### Adequacy of judgment for risk of bias associated with random sequence generation

The majority of the five parent categories of supporting comments had correct judgments by the Cochrane authors. In the category where the method of randomisation was not described, 82% of trials were adequately judged as unclear. For the random number table category, 95% of Cochrane authors correctly judged this as low risk of bias. When the Cochrane authors indicated that there was incomplete or inappropriate randomisation, 87% of such trials were properly judged as having a high risk of bias associated with random sequence generation. For supporting comments describing randomisation via computer/software/internet, 99% of the authors correctly judged it as low risk of bias. For mechanical methods of randomisation, 88% of trials were correctly judged as having a low risk of bias.

Overall, 1,220/10,103 judgments (12%) were not in line with recommendations from the Cochrane Handbook for risk of bias associated with random sequence generation. The highest proportion of mistakes was observed in the parent category ″method of randomisation not described″. For instance, 99% of the trials, where the supporting comment only indicated central randomisation, were inadequately judged as low risk of bias because the method of randomisation was in fact not described. Likewise, 72% of trials that only wrote about baseline balance between groups, 68% of the trials that had only a description of block randomisation or stratification and 51% of trials for which the supporting comment only mentioned that randomisation was done using envelopes were inadequately judged as having a low risk of bias as well (Table 2).

The highest proportion of discrepancies regarding RoB judgment was found in RCTs categorised as having a high risk of bias associated with random sequence generation. There were 365 (3.6%) of RCTs judged as having a high risk of bias for this domain, out of which 101 (28%) were not adequately judged. Of the five parent categories we used, only the category ″incomplete or inappropriate randomisation″ should have been judged as high risk of bias (Table 2).

Among the RCTs judged as having a low risk of bias for random sequence generation (*N* = 4,951, 49%), there were 983 (20%) with inappropriate judgment. Among the five parent categories, low risk of bias should have been associated with the use of a random number table, randomisation using computer/software/internet and with mechanical methods of randomisation.

There were 4,787 RCTs (48%) judged as having an unclear risk of bias for random sequence generation, out of which 136 (3%) were inadequately judged. Out of the five parent categories we used, only comments in the category where the method of randomisation was not described should have been judged as having an unclear risk of bias.

## Discussion

Analysis of judgment and comments about the risk of bias associated with random sequence generation in 10,103 RCTs included in 704 Cochrane reviews indicated that Cochrane authors do not always adhere to recommendations regarding assessment of this particular type of risk of bias and that 12% of judgments for bias associated with this domain were not in line with the recommendations from the Cochrane Handbook. This also implies that one in eight judgments of bias regarding randomisation of participants in Cochrane reviews was not elaborated or that the supporting comment was not detailed enough. The most common discrepancies were observed for the category of trials judged as having high RoB for sequence generation, where 28% of the judgments were not supported with the explanations given in the accompanying supporting comments for the judgment. To the best of our knowledge, this is the first study evaluating rationales for and accuracy of judgments for RoB associated with random sequence generation in Cochrane systematic reviews.

The most frequent discrepancy, according to the parent category, was judging RoB for randomised sequence generation as low when the method of randomisation was not sufficiently described. The most frequent of such supporting comments were related to the mention of central randomisation without further details, baseline balance between the groups where the Cochrane authors assumed that the randomised sequence generation was adequate, self-standing comments about block randomisation or stratification and supporting comments about using envelopes without further details.

Cochrane Handbook explicitly warns: ″*Sometimes trial authors provide some information, but they incompletely define their approach, and do not confirm some random component in the process. For example, authors may state that blocked randomization was used, but the process of selecting the blocks, such as a random number table or a computer random number generator, was not specified. The adequacy of sequence generation should then be classified as unclear*″ [11].

Cochrane has recently proposed *A revised tool to assess risk of bias in randomized trials* (RoB 2.0) [12]. In the RoB 2.0 version, baseline characteristics of participants are featured prominently. The RoB 2.0 uses baseline imbalances to signal problems with the randomisation process, and one of the signalling questions in the RoB domain about randomisation process is ″*Were there baseline imbalances that suggest a problem with the randomization process?*″ [12].

We found 98 instances where random number generation were mentioned without remarks about using a computer, electronic calculators, etc. This category was judged as low risk in 100%, and we left it in the computer parent category, although it did not fulfil strict Cochrane Handbook rules. Otherwise, erroneous levels would rise from 12 to 13% in total and from 20 to 22% for trials judged as having a low risk of bias.

Our findings shed light on the certainty of evidence and reliability of conclusions in Cochrane reviews and a number of meta-epidemiological studies that were based on the RoB assessments from Cochrane reviews. RoB assessment is regularly mentioned in conclusions of Cochrane reviews. It is very common in Cochrane reviews to read that included studies were of high risk of bias, and therefore their results are less reliable. Cochrane authors may conclude that certain findings are based on studies with low RoB, but if one in five of those RoB judgments is flawed, then the conclusions of reviews may be severely compromised. The readers of such reviews get the message that certain recommendations rely on evidence with low RoB and therefore evidence that is more reliable and trustworthy.

In previous studies, authors appear to assume that RoB judgments in Cochrane reviews were apropriately applied and various conclusions that were reached were related to those judgments [5, 13]. A recent study reported that poor reporting and inadequate methods have decreased over time, particularly for sequence generation and allocation concealment, based on the analysis of 20,920 RCTs included in Cochrane reviews [5]. However, our study indicated that this result does not have to be due to better reporting and better methods, but errors in judgment of Cochrane authors.

In our study we found that more than half of the trials included in our sample of Cochrane reviews had unclear risk of bias for generating randomisation sequence, which is in line with a report by Kahan et al., who reported that risk of selection bias is difficult to ascertain in the majority of trials because of poor reporting [14].

Likewise, studies that analysed the association between RoB and effect of interventions could have reached inappropriate conclusions because they trusted the judgment of systematic review authors. Savovic et al. reported that estimates of intervention effects were exaggerated by an average of 11% in clinical trials with inadequate or unclear sequence generation. Their results were based on analysis of 1,973 trials included in 234 meta-analyses [13].

According to the Cochrane Handbook, Cochrane authors can make assumptions but they need to elaborate on them [4]. We considered assumption as the most important factor for such a high proportion of unsupported judgments. We found supporting comments where Cochrane authors just wrote *assumed*, and judged the trial as having a low risk of bias related to randomisation without explaining why they assume that risk is low.

A limitation of our study is a confined time period in which analysed Cochrane reviews were published. However, we analysed a high number of Cochrane reviews, with a high number of included trials, and these Cochrane reviews were published recently. Therefore, we believe that they are representative of the current state of reporting of the analysed domain in Cochrane systematic reviews. Another limitation is a possible human error due to the interpretation of Cochrane Handbook and manual categorisation of automatically extracted text. To prevent this error, every categorisation made by one author was checked by the first author. Also, we acknowledge that our subcategories, created during manual categorisation, are not recognised by the Cochrane RoB tool. However, we pointed them out just to show the most common mistakes that authors make without entering an in-depth analysis. A single comment can sometimes be subcategorised and the decision for sorting a comment into a subcategory was arbitrary because no ranking on this level was defined. However, this did not change the results of this study because the comment remained inside the same parent category.

Furthermore, we acknowledge the possibility that RoB judgments may have been correct based on all the information available to Cochrane authors, but since the authors did not provide enough details in supporting comments, their judgments appear inadequate. In this study we did not attempt to analyse information available in trials included in Cochrane reviews and to compare whether Cochrane authors may have had more information available than shown in a supporting comment. Nevertheless, our study is useful in pointing out inadequate reporting in RoB tables, so that in the future authors using Cochrane RoB tool may pay more attention to detailed justifications of their judgments.

It has already been shown that the Cochrane RoB has low reliability between individual reviewers, as well as across consensus assessments of reviewer pairs [15]. Da Costa et al. argued that the low reliability of the RoB assessment in systematic reviews can have detrimental effects on decision making and healthcare quality [16]. Interventions such as standardised intensive training on RoB assessment were tested, and the results indicate that such interventions can significantly improve the reliability of the Cochrane RoB tool [17]. Apart from author training, other solutions for improving the reliability of RoB judgments would be more stringent peer reviews and editorial assessments of judgments and supporting comments in the Cochrane RoB table.

Additionally, we analysed only Cochrane reviews, which use Cochrane RoB tool. Recent studies have indicated that the RoB instrument did not adequately capture the risk of bias in RCTs [18, 19]. Future studies on Cochrane RoB 2.0 tool are warranted to see how the new tool compares to the current one.

## Conclusion

Authors of Cochrane systematic reviews sometimes make inadequate judgments about the risk of bias related to random sequence generation. Most of these judgments were not elaborated or supported by comments in sufficient detail. Subsequently, this might compromise the reliability of their conclusions. Our results can help authors of both Cochrane and non-Cochrane reviews which use Cochrane RoB tool to avoid making common mistakes when assessing RoB in included trials. Even though systematic reviews are considered the highest level of evidence, our study shows that these types of studies should also be scrutinised to make sure that their every aspect is trustworthy. Interventions for improving the reliability of Cochrane risk of bias assessment should be considered.

## Additional files

Additional file 1: The short module used for data scraping from the webpage. The module uses a formatted spreadsheet containing web addresses of Cochrane reviews. (TXT 38 kb)

Additional file 2: One of the final VBA modules used for extraction of RoB tables from separate spreadsheets. The module includes search code for strings indicating the beginning and the end of RoB tables, as well as code for extraction of original domains and customized domains. All modules were written in VBA originally with .bas extension. Raw code is presented; notice single quotation marks for the exclusion of parts of code while testing. (TXT 1 kb)

## Data Availability

Analyzed data were publicly available information available in the risk of bias tables of Cochrane systematic reviews published in the Cochrane Database of Systematic Reviews (CDSR), http://www.cochranelibrary.com. The datasets used and/or analyzed during the current study are available from the corresponding author on reasonable request.

## Abbreviations

CDSR: The Cochrane Database of Systematic Reviews
CONSORT: Consolidated Standards of Reporting Trials
et al.: et alia: and others
IVRS: Interactive Voice Response System
NRS: Non-Randomized Study
RCT: Randomized Controlled Trial
RoB: Risk of Bias
VBA: Visual Basic for Applications
w/o: without
